# Development and Application of a Rapid Field Detection Technology for DENV-2 Based on the HUDSON Nucleic Acid Extraction-Free/RT-RAA/CRISPR-Cas12a System

**DOI:** 10.3390/v17121579

**Published:** 2025-12-02

**Authors:** Chang Tan, Siyu Xing, Xiaoxue Xie, Xiaoli Chen, Xiaohui Liu, Wenhao Wang, Lifang Liu, Xinyu Zhou, Jiahong Wu, Chunxiao Li

**Affiliations:** 1 Characteristic Key Laboratory of Modern Pathogen Biology, School of Basic Medicine Sciences, Guizhou Medical University, Guian New Area, Guiyang 561113, China; 2 State Key Laboratory of Pathogen and Biosecurity, Beijing 100071, China

**Keywords:** HUDSON, RT-RAA, CRISPR/Cas12a, DENV-2, detection

## Abstract

Dengue fever has become a major global public health challenge due to its rapidly in-creasing incidence. Rapid on-site detection of dengue virus (DENV) is critical for early diagnosis, timely patient isolation, and outbreak control. In this study, dengue virus serotype 2 (DENV-2), the predominant strain circulating in tropical and subtropical regions, was selected as the target pathogen. We established a one-tube rapid detection assay that integrates the HUDSON nucleic acid extraction-free protocol, reverse transcription recombinase-aided amplification (RT-RAA), and CRISPR/Cas12a-mediated trans cleavage activity. The method achieved a detection limit of 1 × 10^2^ copies/μL for simulated infected samples and exhibited no cross-reactivity with other DENV serotypes (DENV-1, DENV-3, DENV-4) or with other arboviruses, including Zika, Japanese encephalitis, yellow fever, and chikungunya viruses. The assay demonstrated high sensitivity and specificity across various sample types, including mosquitoes, rodents, blood, and cultured cells, with results consistent with quantitative PCR (qPCR). Requiring only basic equipment such as a water bath, the system enables on-site detection of DENV-2 within 1 h. This simple, cost-effective, and reliable assay provides a practical tool for field-based DENV-2 surveillance and supports effective public health responses in resource-limited settings.

## 1. Introduction

Dengue fever is a serious mosquito-borne infectious disease that poses a significant global public health threat; the virus continues to circulate in more than 100 countries, putting approximately 4 billion people at risk of infection [1,2,3]. Dengue virus (DENV), a member of the Flavivirus genus, comprises four antigenically distinct serotypes (DENV-1 to DENV-4), among which DENV-2 is considered the most virulent and is the predominant serotype in tropical and subtropical regions [4,5,6]. The virus is transmitted primarily through the bite of Aedes mosquitoes. Following infection, patients may develop symptoms such as fever and thrombocytopenia, and severe cases can progress to life-threatening complications, including plasma leakage and dengue shock syndrome [7,8,9]. Secondary infections with a heterologous serotype are known to markedly increase the risk of severe disease and also complicate vaccine development [10,11]. Currently, no specific antiviral drugs or broadly effective vaccines are available, making early and accurate diagnosis a critical component of dengue surveillance, prevention, and epidemic control [12,13].

The early symptoms of dengue fever are non-specific and can easily be confused with those of chikungunya fever, Zika virus disease, and other conditions—thus making diagnosis challenging [14]. Existing detection methods, including virus isolation, ELISA, and RT-PCR, are contingent on specialized equipment and personnel, which hinders their feasibility at the grassroots level [15,16]. The CRISPR/Cas system, an immune mechanism found in prokaryotic organisms, has been adapted for use in a novel nucleic acid detection tool. The Cas12a protein, guided by crRNA, has been demonstrated to cleave double-stranded DNA with a high degree of specificity, and subsequently induces non-specific single-stranded DNA trans-cleavage. This property facilitates a remarkably sensitive detection mechanism [17,18,19]. However, most CRISPR-based detection strategies still require preliminary nucleic acid amplification steps such as PCR, which depend on sophisticated laboratory equipment and are therefore unsuitable for field deployment [20,21]. To meet the growing demands of point-of-care testing (POCT), isothermal nucleic acid amplification technologies (iNAATs) have gained widespread attention, with recombinase polymerase amplification (RPA), recombinase-aided amplification (RAA), and enzyme recombination amplification (ERA) being among the most representative systems. RPA relies on phage-derived recombinases [22] and therefore requires a highly stable reaction system. ERA employs engineered enzymes to modulate reaction kinetics, whereas RAA utilizes recombinant enzymes of bacterial or fungal origin. In RAA, the coordinated activity of the recombinase, single-strand binding protein (SSB), and DNA polymerase enables rapid strand displacement amplification at 37–42 °C without the need for a high-temperature denaturation step [23]. Owing to its low-temperature operation, rapid amplification, and user-friendly workflow, RAA has been widely integrated into freeze-dried reagent formats and portable point-of-care testing (POCT) platforms [24,25,26]. In recent years, the combination of RT-RAA with CRISPR/Cas systems has further enabled the successful detection of multiple viral pathogens, including SARS-CoV-2 [27], nervous necrosis virus (NNV) [28], and maize chlorotic mottle virus (MCMV) [29].

Among the various CRISPR systems, Cas12a is a double-stranded DNA (dsDNA) targeting nuclease that retains robust activity at low temperatures of approximately 37 °C [30], making it highly compatible with the optimal reaction conditions of RAA. Moreover, the combination of RAAs low tolerance for primer–template mismatches and the high single-nucleotide discrimination capability of Cas12a–crRNA provides a unique advantage, enabling dual-level specificity for viral sequence and serotype identification [31]. In contrast, Cas12b and Cas13a are less suitable for low-temperature, rapid detection scenarios due to their higher optimal reaction temperatures and more complex assay workflows [32,33]. Although RAA has been employed for rapid visual detection of dengue virus (e.g., RT-RAA-LFD) [34], systematic studies focusing on DENV-2 serotype-specific detection platforms that use RAA as the core amplification module and are tightly integrated with Cas12a remain limited.

Building on these findings, this study established an integrated RAA–Cas12a detection platform. To address the contamination risks associated with opening reaction tubes and the laborious nucleic acid extraction procedures required by conventional two-step workflows, we further developed a single-tube “one-pot” system. In this design, the RT-RAA reaction mixture is placed at the bottom of the tube, while the CRISPR/Cas12a components are pre-loaded into the lid. After amplification, a brief centrifugation initiates the CRISPR detection process, eliminating the need to open the tube and thereby reducing the risk of aerosol contamination. Additionally, this study incorporates the HUDSON (Heating Unextracted Diagnostic Samples to Obliterate Nucleases) sample-processing method, first introduced by Myhrvold et al. [35] in 2018. HUDSON uses TCEP/EDTA-assisted heat treatment to directly lyse viral particles and inactivate RNases, enabling the use of unpurified samples for subsequent amplification and detection. This system integrates sample processing, nucleic acid amplification, and CRISPR-based detection into a single cohesive platform, providing a simple, rapid, and field-deployable point-of-care testing solution for dengue virus and mosquito vector samples.

## 2. Materials and Methods

### 2.1. Virus and Cells

The Dengue virus serotype 2(DENV-2, NGC strain GenBank accession no. KM204118.1), Zika virus (ZIKV, SZ01 strain, GenBank accession no. KU866423), Japanese encephalitis virus (JEV, SA-14-14-2 strain, GenBank accession no. JN604986.1), Yellow fever virus (YFV, 17D-204 strain, GenBank accession no. KF769015.1), and Chikungunya virus (CHIKV, QZ0823 strain, GenBank accession no. MH400249) used in this study were previously obtained and stored at –80 °C in the State Key Laboratory of Pathogens and Biosecurity.

The nucleic acids of Dengue virus serotype 1 (DENV-1, JH545 strain, GenBank accession no. OP919589.1), Dengue virus serotype 3 (DENV-3, 01442/00 strain, GenBank accession no. MW946892.1), and Dengue virus serotype 4 (DENV-4, YGH_16_18 consensus strain, GenBank accession no. MW788998.1) were kindly provided by the Nanjing Bioengineering (Gene) Technology Center for Medicines (Nanjing, China). All viral nucleic acid samples were stored at –80 °C.

BHK-21 cells and Vero cells were obtained from the State Key Laboratory of Pathogens and Biosecurity. The cells were maintained in Dulbecco’s modified Eagle’s Medium (DMEM; Thermo Fisher Scientific, Waltham, MA, USA) supplemented with 10% fetal bovine serum (FBS; Gibco, Carlsbad, CA, USA) and cultured at 37 °C in a 5% CO_2_ incubator (Thermo Fisher Scientific, Waltham, MA, USA).

### 2.2. Conventional Nucleic Acid Extraction and HUDSON Nucleic Acid Extraction

Conventional nucleic acid extraction was performed using the commercial QIAamp RNA Extraction Kit (Qiagen, Hilden, Germany) to isolate viral nucleic acids from DENV-2 virus suspensions.

The HUDSON nucleic acid extraction process involved adding TCEP (MackLin, Shanghai, China) and EDTA (Thermo Fisher Scientific, Waltham, MA, USA) to the DENV-2 virus suspension at final concentrations of 100 mM and 1 mM, respectively. The mixture was then incubated at 42 °C for 20 min, and subsequently at 64 °C for 5 min in a PCR instrument (Thermo Fisher Scientific, Waltham, MA, USA) to release nucleic acids through heat-induced denaturation.

A seven-step serial dilution of the DENV-2 virus suspension was prepared using nuclease-free water (Zoman, Beijing, China), starting from 1 × 10^8^ copies/μL and diluted at a 1:9 ratio down to 1 × 10^1^ copies/μL. Nucleic acids from each dilution were released using both the commercial extraction kit and the HUDSON method. The extracted nucleic acids were subsequently amplified using the HiScript II One Step qRT-PCR SYBR Green Kit (Vazyme, Nanjing, China), and the Ct values were determined on an Applied Biosystems QuantStudio 7 Real-Time PCR (Thermo Fisher Scientific, Waltham, MA, USA). The total reaction volume was 20 μL and consisted of 10 μL of 2× One Step SYBR Green Mix, 1 μL of One Step SYBR Green Enzyme Mix, 0.2 μL of 50× ROX Reference Dye, 0.4 μL of the forward primer (10 μM), 0.4 μL of the reverse primer (10 μM), and 2 μL of template RNA. The remaining volume (5.8 μL) was filled with nuclease-free water to reach a final volume of 20 μL. The primer sequences were identical to those used in the RT-RAA DENV-2 amplification system and targeted the E1 coding region of dengue virus serotype 2 (DENV-2). The detailed qRT-PCR protocol is provided in Table 1. The performance of the two nucleic acid extraction methods was then compared.

### 2.3. One-Pot RT-RAA-CRISPR/Cas12a DENV-2 Detection System

The Reverse Transcription Recombinase-Aided Amplification (RT-RAA) reaction was performed using a commercial isothermal RNA rapid amplification kit (Qitian Gene Biotech, Wuxi, China). Each reaction was carried out in a final volume of 25 μL. The reaction mixture contained 12.5 μL of Buffer V, 1 μL each of the F1 and R1 primers (10 μM), 2.5 μL of magnesium acetate, 7 μL of nuclease-free water, and 1 μL of viral nucleic acid. The reaction was incubated at 39 °C for 25 min, and the amplification products were verified by gel electrophoresis.

For CRISPR/Cas12a detection, a 10 μL reaction mixture was prepared in the tube cap. The mixture contained 3.5 μL of HOLMES buffer (10×, ToLo Biotech, Shanghai, China), 1 μL of LbCas12a (ToLo Biotech, Shanghai, China), 1 μL of ssDNA reporter (Bio-Lifesci, Guangzhou, China), 1 μL of crRNA (EditGene, Guangzhou, China), and 3.5 μL of nuclease-free water (Zoman, Beijing, China). After amplification, the CRISPR/Cas12a reaction mixture was spun down into the RT-RAA reaction using a mini high-speed centrifuge (Yooning Instrument, Hangzhou, China). The reaction was then incubated at 39 °C for 20 min in an Applied Biosystems QuantStudio 7 real-time PCR system (Thermo Fisher Scientific, Waltham, MA, USA), during which dynamic FAM fluorescence signals were recorded at 30-s intervals.

### 2.4. Design of DENV-2-Specific RT-RAA Amplification Detection Primers and Cas12a crRNA

Multiple sets of DENV-specific primers for RT-RAA detection were designed to target six coding regions of the DENV-2 genome (C1/E1/NS1/NS2/NS3/NS5), comprising six forward primers and six reverse primers, all synthesized by Sango Biotech (Shanghai, China). In addition, six crRNAs targeting the corresponding genomic regions were designed using the Benchling online platform (Benchling, San Francisco, CA, USA; https://benchling.com (accessed on 23 January 2025)), based on the LbCas12a PAM sequence (TTTV). All crRNAs were synthesized by EditGene (Guangzhou, China). The complete sequences of the primers and crRNAs are listed in Table 2 and Table 3, respectively.

### 2.5. Effects of Amplification Time, Temperature, Probe Concentration, and Other Reaction Conditions

Using viral nucleic acid (1 × 10^8^ copies/μL) as the template and following a single variable experimental design, fluorescence intensity was measured under different reaction conditions. These conditions included RT-RAA amplification times (15, 20, 25, and 30 min), CRISPR/Cas12a detection system reaction temperatures (35, 37, 39, 41, and 43 °C), Cas12a concentrations (125, 250, 500, and 1000 nM), Cas12a/crRNA molar ratios (2:1, 1:1, 1:2, and 4:1), and ssDNA probe concentrations (1, 2, 3, 4, 5, 6, 7, 8, 9, and 10 μM).

### 2.6. Sensitivity and Specificity Analysis

A DENV-2 virus suspension at 1 × 10^8^ copies/μL was serially diluted seven times using nuclease-free water at a 1:9 ratio to obtain dilutions down to 1 × 10^1^ copies/μL. Viral suspensions at each dilution level were subjected to nucleic acid extraction and subsequently used as templates for RT-RAA amplification under the conditions described above. Following amplification, CRISPR/Cas12a detection was performed, with nuclease-free water serving as the negative control. The sensitivity of the assay was assessed based on fluorescence intensity measurements.

The amplification and detection process was carried out under the reaction conditions described above, using nucleic acids from other arboviruses, including ZIKV, JEV, YFV, and CHIKV, as well as from the other three DENV serotypes, as templates. Nuclease-free water was used as the negative control to assess the specificity of the assay.

### 2.7. Construction and Detection of Virus-Infected Simulation Samples

In this study, a series of DENV-2–infected materials were prepared. First, the BHK-21 and Vero cells were inoculated with DENV-2 and incubated for 72 h at 37 °C in a 5% CO_2_ atmosphere. The culture supernatant was collected after a centrifugation process at 3000 rpm (1000× *g*) for 5 min using a Sartorius centrifuge (Göttingen, Germany), and the viral RNA extracted from the supernatant was used as the cell infection sample. Second, 6-week-old SPF BALB/c mice (Charles River, Beijing, China) were inoculated intramuscularly in the thigh with 500 μL of DENV-2 suspension using a sterile 1 mL syringe (WEGO, Weihai, China). Blood samples were collected from the orbital venous plexus using a capillary tube (Sutter Instrument, Novato, CA, USA) followed by centrifugation at 3000 rpm (1000× *g*) for 10 min to obtain serum. The extracted serum RNA was used as the mammalian infection sample. Third, laboratory-infected Aedes aegypti (Xishuangbanna strain) and Aedes albopictus (Guangzhou strain) mosquitoes were mixed with uninfected mosquitoes at ratios of 1:9, 1:19, 1:29, 1:39, and 1:49. Each mosquito pool was homogenized and centrifuged at 12,000 rpm for 10 min. The resulting supernatants were collected for RNA extraction and served as simulated field-collected mosquito infection samples. All RNA extraction procedures were performed under identical conditions, followed by RT-RAA amplification. The amplified products were subsequently analyzed using the LFA test strip (Bio-Lifesci, Guangzhou, China) in combination with the CRISPR-Cas12a system. In parallel, each sample was quantitatively analyzed using the HiScript II One Step qRT-PCR Probe Kit with an RT-qPCR instrument.

## 3. Results

### 3.1. Establishment of the Nucleic Acid Extraction Method Based on the HUDSON Protocol

**Figure 1:**
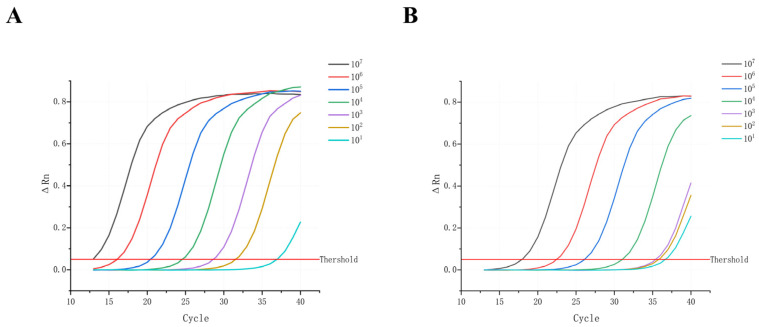
Comparison of nucleic acid extraction efficiency between the commercial kit and the HUDSON method. (**A**) RT-qPCR detection results for nucleic acids extracted using the commercial kit method. (**B**) RT-qPCR detection results for nucleic acids released using the HUDSON method.

This study conducted a parallel comparison of the HUDSON method and a commercial extraction kit. The RT-qPCR results (Figure 1A,B) show that the HUDSON method effectively releases viral nucleic acids. Although the HUDSON method exhibited slightly lower sensitivity than the commercial kit, the Ct values and the amplification obtained by the two methods were highly consistent, indicating no meaningful difference in overall extraction efficiency. Therefore, the HUDSON method was selected for nucleic acid extraction in all subsequent experiments.

### 3.2. Establishment of a One-Pot RT-RAA-CRISPR/Cas12a Assay for DENV-2 Detection

**Figure 2:**
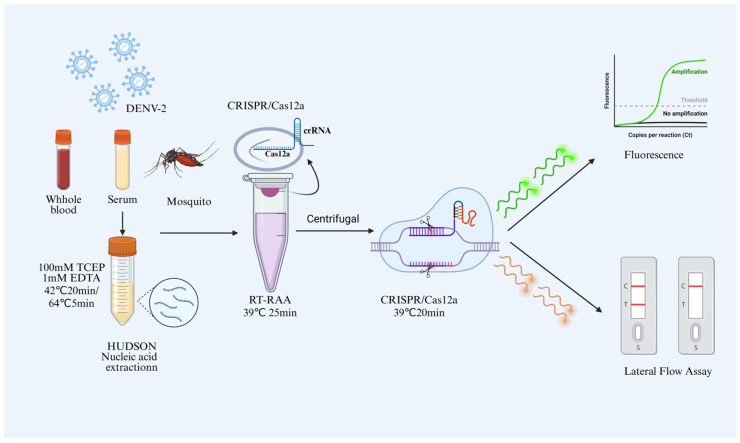
Workflow diagram of the one-pot assay. Color elements in the schematic are used solely for illustration and to distinguish different components; they do not represent experimental differences.

The workflow of the RT-RAA CRISPR/Cas12a assay for DENV-2 detection is shown in Figure 2. Briefly, nucleic acids were first extracted from different types of DENV-2-infected samples using the HUDSON method and then used as templates for RT-RAA amplification. The amplification reaction was performed at 39 °C for 25 min at the bottom of the tube, while the CRISPR/Cas12a detection mixture was prepared separately in the tube cap. After RT-RAA amplification was complete, the two components were combined by brief centrifugation and incubated at 39 °C for an additional 20 min. Detection was achieved using an FAM-labelled single-stranded DNA reporter. Upon recognition of the target nucleic acid by a specific crRNA, the CRISPR/Cas12a complex became activated and cleaved the reporter, generating a measurable fluorescent signal. This closed-tube format enables contamination-free and universal DENV-2 detection without the need for tube opening or additional manipulation. Furthermore, a lateral flow detection (LFD) platform was developed, which also exhibited excellent performance for detecting a wide range of DENV-2–infected samples.

### 3.3. Validation of the RT-RAA-Cas12a Fluorescence Reaction System and Selection of RT-RAA Primers and crRNA

**Figure 3:**
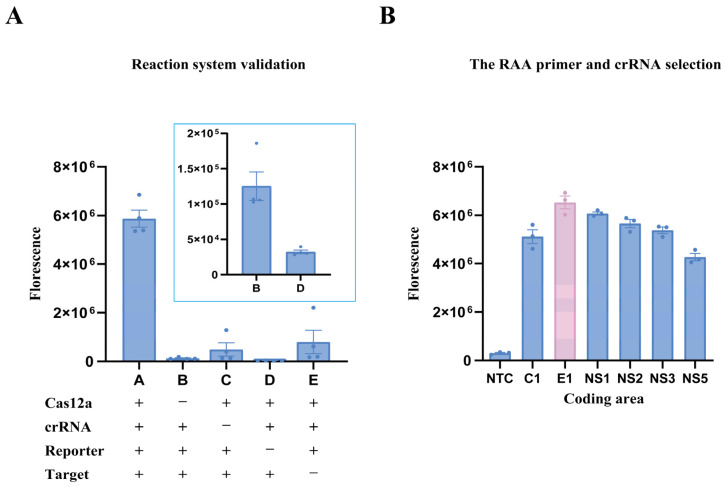
Validation of the RT-RAA-Cas12a fluorescence reaction system and reaction results for six loci within the DENV-2 genome. (**A**) Component integrity validation of the DENV-2 RT-RAA-Cas12a fluorescence system. Fluorescence signals appeared only when all components (Cas12a, crRNA, ssDNA-FQ reporter, and target amplicon) were present, confirming the necessity of complete system integrity for signal generation. Fluorescence output was quantified under reaction conditions in which individual components were systematically omitted to assess their necessity for Cas12a activation. Reaction groups A–E correspond to the following compositions: A, complete reaction containing Cas12a, crRNA, reporter, and target; B, absence of Cas12a; C, absence of crRNA; D, absence of reporter; E, absence of target. (**B**) Comparison of six DENV-2 genomic targets in the RT-RAA-Cas12a assay. Among the C, E, NS1, NS2, NS3, and NS5 targets, the E-gene primer–crRNA pair yielded the strongest fluorescence, indicating the highest detection sensitivity and serving as the optimal target for subsequent analyses.

To evaluate the performance of the DENV-2 RT-RAA-Cas12a fluorescence detection system and identify the optimal target sequence, two sets of experiments were designed: component integrity validation and multi-target sequence comparison. These experiments aimed to verify system specificity and maximize detection sensitivity. In the first experiment, individual components including Cas12a protein, crRNA, the ssDNA-FQ reporter probe, and the target amplification product were systematically omitted to examine their impact on fluorescence output. In the second experiment, Cas12a-mediated fluorescence detection was conducted for 20 min under identical conditions using primer–crRNA pairs targeting six conserved regions (C, E, NS1, NS2, NS3, and NS5), based on previously designed RT-RAA primers and 5′-TTTN PAM crRNAs. As shown in Figure 3A, a marked increase in fluorescence signal occurred only when all components—Cas12a protein, crRNA, ssDNA-FQ reporter probe, and the target amplicon—were present, indicating that complete system integrity is required for signal generation. Among the six target regions tested, the primer–crRNA pair targeting the E gene produced the strongest fluorescence signal, significantly surpassing the other five groups (Figure 3B). Therefore, the E-gene target was selected for all subsequent experiments.

### 3.4. Optimization of the One-Pot RT-RAA-CRISPR/Cas12a Detection Reaction Conditions

**Figure 4:**
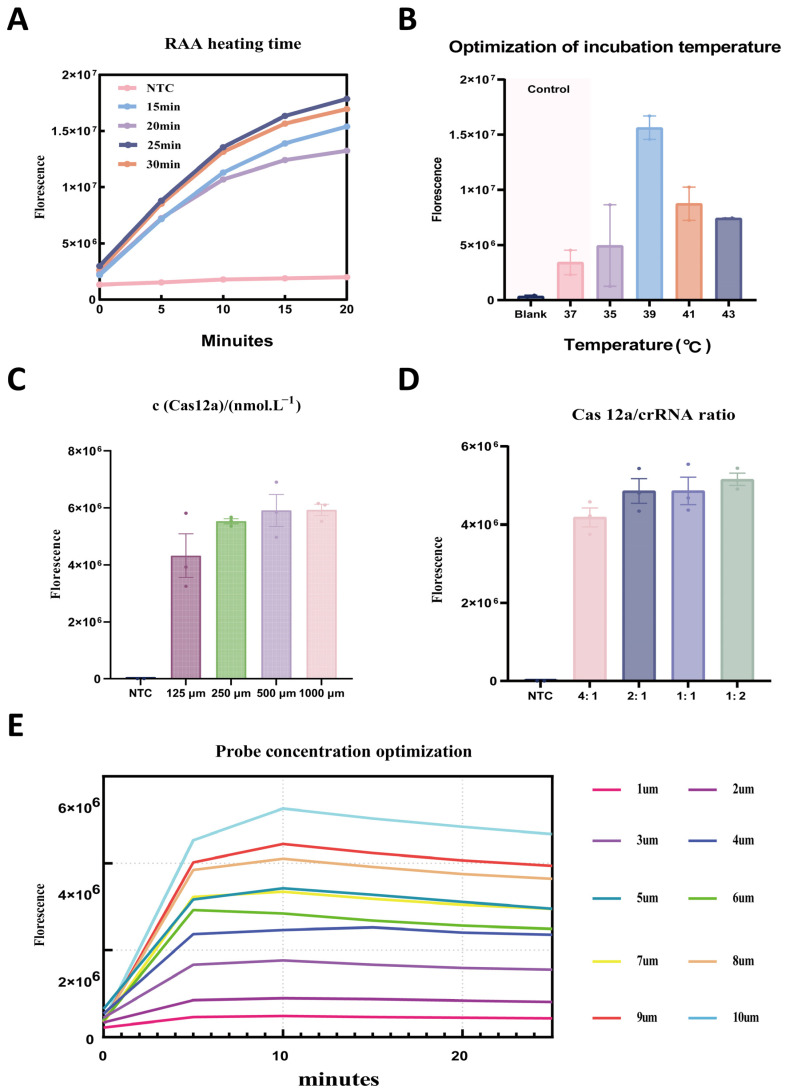
Optimization of a Single-Pot RAA CRISPR/Cas12a System for the Detection of DENV-2. (**A**) Optimization of RT-RAA incubation time for the DENV-2 RT-RAA-Cas12a fluorescence assay. (**B**) Effect of CRISPR reaction temperature on Cas12a cleavage activity. (**C**) Effect of Cas12a concentration on fluorescence signal intensity. (**D**) Effect of the Cas12a-to-crRNA molar ratio on fluorescence signal. (**E**) Optimization of ssDNA-FQ probe concentration.

To improve the sensitivity and signal-to-noise ratio of the DENV-2 RT-RAA-Cas12a fluorescence detection system while minimizing reaction time and reagent consumption and maintaining maximal fluorescence output, five key parameters were systematically optimized using a single-factor rotation strategy: RT-RAA incubation time, CRISPR reaction temperature, Cas12a concentration, Cas12a-to-crRNA molar ratio, and ssDNA probe concentration. Endpoint fluorescence intensity was measured after a 20-minCRISPR/Cas12a reaction. As shown in Figure 4A, fluorescence intensity reached a plateau at an RT-RAA incubation time of 25 min, with no further increase upon extending the reaction time. Cas12a exhibited maximal cleavage activity at 39 °C, whereas either higher or lower temperatures resulted in decreased fluorescence output (Figure 4B). The strongest fluorescence signal was obtained at a final Cas12a concentration of 500 nM and a Cas12a-to-crRNA molar ratio of 1:2, while deviations from this ratio resulted in reduced signal intensity (Figure 4C,D). Additionally, the ssDNA-FQ probe produced saturated fluorescence at a concentration of 10 μM; further increases in probe concentration elevated background fluorescence without improving signal intensity (Figure 4E).

### 3.5. Sensitivity and Specificity Analysis of the One-Pot RT-RAA-CRISPR/Cas12a Detection Method

**Figure 5:**
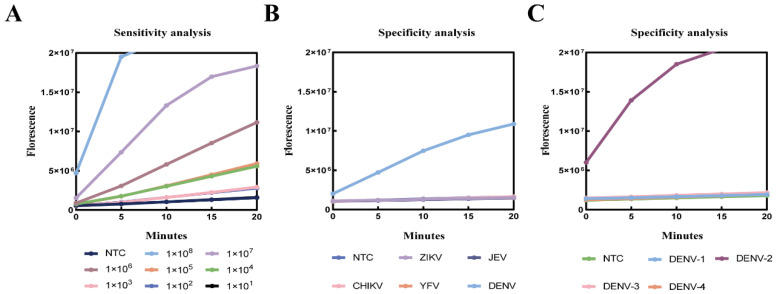
Test Results for Sensitivity and Specificity of the CRISPR/Cas12a System. (**A**) Sensitivity analysis of the DENV-2 RT-RAA-Cas12a fluorescence detection system. (**B**) Cross-reactivity analysis with other arboviruses. (**C**) Specificity evaluation of the DENV-2 RT-RAA-Cas12a fluorescence detection system against different DENV serotypes.

To assess the sensitivity and specificity of the DENV-2 RT-RAA-Cas12a fluorescence detection system, and to verify its ability to accurately identify DENV-2 in mixed arbovirus samples without cross-reactivity, gradient-diluted DENV-2 nucleic acid samples (10^8^–10^1^ copies/μL) were tested alongside nucleic acids from other arboviruses, including DENV-1/3/4, ZIKV, CHIKV, YFV, and JEV under the optimized reaction conditions. As shown in Figure 5A, the cleavage kinetics of Cas12a targeting the E gene progressively slowed as the DENV-2 copy number decreased, and the detection limit of the assay was determined to be 1 × 10^2^ copies/μL. In addition, DENV-2 nucleic acids were accurately distinguished from those of other DENV serotypes and other arboviruses, with no detectable cross-reactivity (Figure 5B,C), indicating that the assay exhibits 100% specificity for DENV-2.

### 3.6. Detection of Simulated Virus-Infected Samples

**Figure 6:**
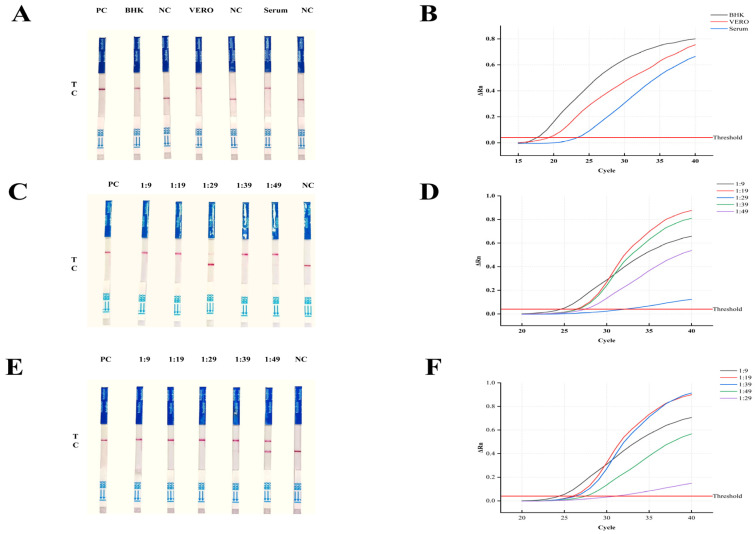
Target RNA detection results obtained using the RT-RAA/CRISPR and qPCR assays. (**A**,**B**) Test results for cell samples. (**A**) RT-RAA/CRISPR Lateral Flow Test Strips. PC: Positive control; BHK: BHK-21 cell; Vero: Vero cell; Serum: Serum sample; NC: Negative control. (**B**) qPCR amplification curve for the corresponding sample. (**C**,**D**) Test results for Aedes aegypti Samples. (**C**) RT-RAA/CRISPR test strips (samples 1:9–1:49) along with positive and negative controls. (**D**) qPCR amplification curve for the corresponding sample. (**E**,**F**) Test results for Aedes albopictus samples. (**E**) RT-RAA/CRISPR test strips (samples 1:9–1:49) along with positive and negative controls. (**F**) qPCR amplification curve for the corresponding sample. The blue arrows and the “MAX” label indicate the correct sample-loading end of the strip and mark the maximum immersion depth during dipping.

The objective of this study is to evaluate the applicability of the RT-RAA/CRISPR detection method across multiple sample types and its potential use in field vector surveillance. Cell samples (BHK, Vero), serum samples, and mosquito samples (Aedes aegypti and Aedes albopictus) were analyzed and compared with qPCR quantification results (Figure 6A–F). The corresponding copy numbers are summarized in Table 4. All test samples yielded positive signals, whereas the negative controls showed no detectable fluorescence. In general, strong positive bands corresponded to higher viral loads (10^3^–10^5^ copies), while weak positive bands were primarily observed in samples with low viral copy numbers (<10^3^). These findings presented herein demonstrate that the RT-RAA/CRISPR assay reliably detects target RNA in both cellular and mosquito samples. Its sensitivity and specificity are comparable to those of qPCR, while offering additional advantages such as operational simplicity and intuitive result interpretation. Overall, the system shows strong potential for field-based vector surveillance applications.

## 4. Discussion

Dengue virus (DENV) is a mosquito-borne flavivirus and one of the most prevalent vector-borne viral pathogens in tropical and subtropical regions [36], posing a substantial threat to global public health [37]. Given the current lack of specific antiviral therapies and broadly effective vaccines, the development of rapid and accurate early detection technologies for DENV is essential for epidemic prevention and for strengthening public health responses.

**Figure 7:**
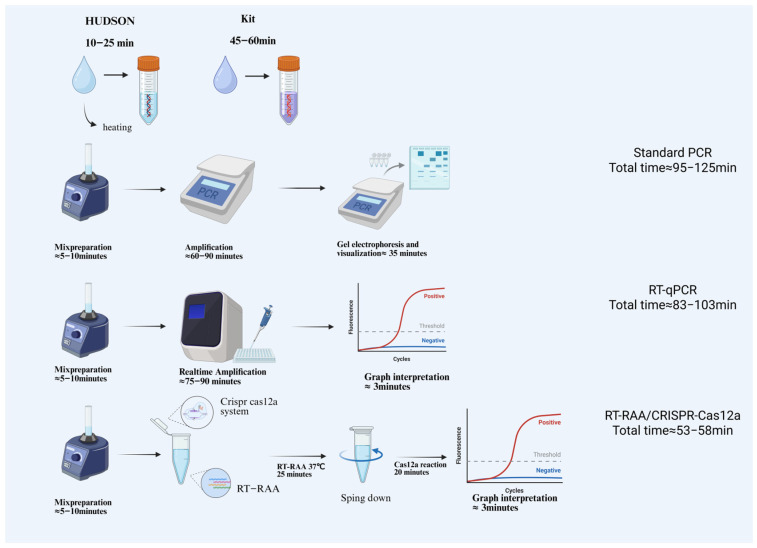
Comparison of HUDSON with Kit-Based PCR/RT-qPCR and One-Pot RT-RAA-CRISPR/Cas12a Detection Workflows. The schematic illustrates the major steps and overall assay time for three nucleic-acid detection workflows. The time required for sample processing—using either HUDSON or a commercial kit—is shown separately to highlight differences in pretreatment duration. After sample preparation, each detection method proceeds through its specific workflow, with the total assay time displayed on the right. Standard PCR includes reaction mixture preparation, thermal cycling amplification, and gel electrophoresis. RT-qPCR involves reaction setup, real-time amplification, and automated fluorescence interpretation. The RT-RAA/CRISPR-Cas12a assay comprises reaction preparation, isothermal amplification, brief centrifugation, and Cas12a-based fluorescence detection. Colors are for visualization only and have no experimental meaning.

In recent years, CRISPR-Cas-based nucleic acid detection platforms have shown substantial potential for pathogen diagnostics [38]. In this study, we successfully established an RAA-CRISPR-Cas12a assay for the rapid detection of DENV-2, achieving a sensitivity of 100 copies/μL. This detection limit is consistent with that of the one-step DENV assay reported by Zhang et al. [16] and surpasses that of conventional PCR methods, which typically detect 10^2^–10^3^ copies/μL. These findings highlight the promising applicability of this assay for field-based dengue surveillance. After system optimization and performance evaluation, several clear advantages of this approach were demonstrated. First, the assay allows visual result interpretation using lateral flow test strips, eliminating the need for complex instrumentation or specialized personnel. Second, unlike the strategy used by Wang et al. [39], who employed RAA or PCR alone, our method integrates RAA amplification with CRISPR detection, leveraging the high specificity of Cas12a. This design minimizes the loss of trace nucleic acids during handling and thereby enhances detection accuracy. Third, the entire workflow operates as a closed, single-tube reaction. In contrast to methods reported by Kellner et al. [40] for ZIKV detection and by Ma et al. [41] for gonococcal detection, which require opening the reaction tube during amplification, this closed-tube format markedly reduces the risk of aerosol contamination. This study also integrated the HUDSON nucleic acid release procedure with RT-RAA amplification and CRISPR/Cas12a detection into a unified diagnostic system. The HUDSON method releases nucleic acids by heating small sample volumes mixed with TCEP and EDTA (for example, serum samples at 42 °C for 20 min, followed by 64 °C for 5 min, and urine samples at 95 °C for 10 min). Compared with traditional kit-based extraction methods, which generally require over 140 μL of sample, involve cumbersome procedures, and incur high costs, HUDSON is simple, rapid, and cost-effective. Although reverse transcription quantitative PCR (RT-qPCR) remains the diagnostic gold standard, it requires expensive instrumentation [42] and well-equipped laboratories [43] and is typically time-consuming. By incorporating the HUDSON method, our assay reduced the overall sample-to-result time to approximately 55–70 min, offering clear advantages in both cost and turnaround time (Figure 7). Regarding specificity, our method was compared with existing commercial assays, such as PanBio Capture IgM ELISA, which has a reported sensitivity and specificity of 86% and 87%, respectively [44]. In contrast our system exhibited no cross-reactivity when tested against multiple arboviruses, including DENV-1/3/4, ZIKV, JEV, CHIKV, and YFV, effectively avoiding false-positive results caused by serological or genetic cross-reactivity and thereby substantially enhancing diagnostic specificity. The results from simulated sample testing indicate that this method exhibits high sensitivity and robust operability under conditions representative of field surveillance. When combined with lateral flow strip-based visual readouts, it is particularly well suited for vector monitoring of dengue fever and for on-site prevention and control efforts in resource-limited settings. In addition, the method is highly economical, with an estimated cost of only USD 4.53 per test, making it feasible for large-scale deployment. In summary, the nucleic acid-free/RT-RAA/CRISPR-Cas12a–based on-site rapid detection method for DENV-2, developed in this study, offers the combined advantages of speed, accuracy, and cost-effectiveness, making it suitable as a promising tool for large-scale screening and field vector surveillance applications.

## 5. Conclusions

In this study, we developed a rapid point-of-care detection technology system for DENV-2 based on the HUDSON nucleic acid-free procedure, RT-RAA amplification, and CRISPR-Cas12a detection. The system demonstrated high specificity and sensitivity for DENV-2, with no false-positive results observed. Moreover, the assay requires only minimal instrumentation, making it particularly suitable for large-scale population screening and field-based vector surveillance. Overall, this technology provides an efficient tool for the early diagnosis of DENV-2 infection and offers reliable technical support for dengue surveillance, prevention, and public health response efforts.

## Figures and Tables

**Table 1:** The One Step qRT-PCR program, run as follows.

Stage 1	Reverse Transcription	Rep:1	50 °C	3 min
Stage 2	Initial Denaturation	Rep:1	95 °C	30 s
Stage 3	Amplification	Rep:40	95 °C	10 s
			60 °C	30 s
Stage 4	MeltCurve Analysis	Rep:1	95 °C	15 s
			60 °C	60 s
			95 °C	15 s

**Table 2:** The RAA primers used in this study include six forward primers (F) and six reverse primers (R).

Primer	Sequence (5′-3′)
DENV C-F1	aaaggcgagaaatacgcctttcaatatgct
DENV C-R1	tctgcgtctcctgttcaagatgttcagcat
DENV E-F1	tttcaggaggaagctgggttgacatagtcttagaa
DENV E-R1	tcacaatgcctccttttccaaataatccac
DENV NS1-F1	aacaaagaactgaagtgtggcagtgggatt
DENV NS1-R1	ttctgatagaatgtgattcaattctggtgt
DENV NS2-F1	gggaagatcaggcagagatatcaggaagca
DENV NS2-R1	tcttcacttcccacaggtaccatgctgctg
DENV NS3-F1	gattgaaccatcatgggcggacgttaagaa
DENV NS3-R1	atatgctccactccttgtaacaacaccatt
DENV NS5 -F1	aacaaggtggtgcgtgtgcaaagaccaacaccaag
DENV NS5-R1	aatcatctccactgatggccattcttgataacctt

**Table 3:** The six sets of Cas12a crRNAs used in this study.

Primer	Sequence (5′-3′)
C crRNA	cuaacaaucccaccaacagcaggg
E crRNA	aacUgaUaaaaacagaagccaaa
NS1 crRNA	aucacagacaacgugcacaca
NS2 crRNA	acuaggagucuugggaauggca
NS3 crRNA	uccuggaaccucaggaucucc
NS5 crRNA	accaauauggaagcccaacuaa

**Table 4:** Comparison of RT-RAA/CRISPR detection results and qPCR copy numbers across different sample types.

Sample Type	RT-RAA-CRISPR/Cas12a	qPCR (Copies/μL)
BHK cells	Positive	275,422.87
Vero cells	Positive	141,253.75
Serum	Positive	31,622.78
Aedes aegypti (1:9)	Positive	5754
Aedes aegypti (1:19)	Positive	2949
Aedes aegypti (1:29)	Positive	308
Aedes aegypti (1:39)	Positive	1096
Aedes aegypti (1:49)	Positive	734
Aedes albopictus (1:9)	Positive	5495
Aedes albopictus (1:19)	Positive	2691
Aedes albopictus (1:29)	Positive	2454
Aedes albopictus (1:39)	Positive	831
Aedes albopictus (1:49)	Positive	504

## Data Availability

All data generated or analyzed during this study are included in this published article.
